# Elucidating the genetic basis of social interaction and isolation

**DOI:** 10.1038/s41467-018-04930-1

**Published:** 2018-07-03

**Authors:** Felix R. Day, Ken K. Ong, John R. B. Perry

**Affiliations:** MRC Epidemiology Unit, Box 285 Institute of Metabolic Science, Cambridge Biomedical Campus, University of Cambridge School of Clinical Medicine, Cambridge, CB2 0QQ UK

## Abstract

The negative impacts of social isolation and loneliness on health are well documented. However, little is known about their possible biological determinants. In up to 452,302 UK Biobank study participants, we perform genome-wide association study analyses for loneliness and regular participation in social activities. We identify 15 genomic loci (*P* < 5 × 10^−8^) for loneliness, and demonstrate a likely causal association between adiposity and increased susceptibility to loneliness and depressive symptoms. Further loci were identified for regular attendance at a sports club or gym (*N* = 6 loci), pub or social club (*N* = 13) or religious group (*N* = 18). Across these traits there was strong enrichment for genes expressed in brain regions that control emotional expression and behaviour. We demonstrate aetiological mechanisms specific to each trait, in addition to identifying loci that are pleiotropic across multiple complex traits. Further study of these traits may identify novel modifiable risk factors associated with social withdrawal and isolation.

## Abstract (web summary)

Little is known about the genetic determinants of social isolation and loneliness despite their well-established importance for health. Here, using multi-trait GWAS, Day et al. identify 15 genomic loci for loneliness and further show a bidirectional causal relationship between BMI and loneliness by MR.

## Introduction

The quality and quantity of social interactions are well-established factors in health and disease, particularly amongst the elderly^1^. Around one in four people over the age of 65 in the UK suffer from loneliness^2^, a social state strongly associated with increased all-cause mortality^3,4^. The magnitude of this effect is comparable to smoking, and exceeds other well established mortality risk factors such as obesity and physical activity^5^. Whilst a range of socioeconomic, behavioural and physiological factors have been associated with loneliness^4^, the causal nature of these relationships is often unclear. For example, does the onset of depressive symptoms and cognitive decline cause withdrawal from social engagement, or is this a consequence of the discrepancy between preferred and experienced social relations. To address this question, and provide insights into the potential biological mechanisms that contribute to loneliness, we identify associated genetic variants in the UK Biobank study^3,6^. Several questions related to loneliness and social isolation were  included in the UK Biobank self-report questionnaire, and these were recently reported to predict all-cause mortality^3^. Under the hypothesis that some forms of social interaction may have unique biological determinants, we also aim to identify genetic variants associated with regular participation in each of three social activities recorded in UK Biobank—sports club or gym, pub or social club, and religious group. Although previous studies have identified a heritable component to loneliness^7^, none has been sufficiently powered to identify individual genetic determinants. Here, we report the identification of ~50 robustly associated genetic variants for loneliness and social interaction. These data highlight shared genetic architecture between loneliness and a range of complex traits, including a causal relationship (based on Mendelian randomisation) between body size and loneliness/depressive symptoms.

## Results and Discussion

### Genetic discovery for loneliness

**Fig. 1:**
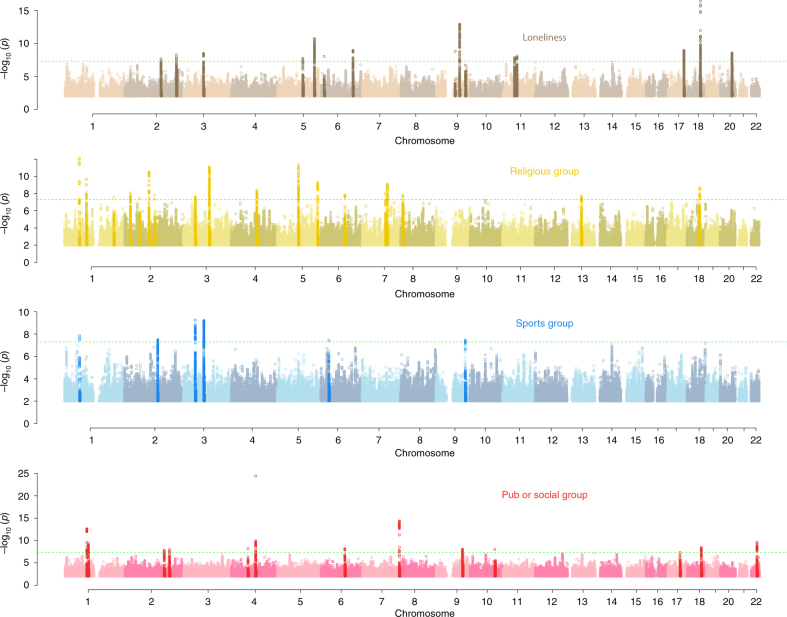
Manhattan plots for the four social interaction  traits. In each case the green horizontal  dotted line denotes genome-wide significance, and the highlighted SNPs are in loci within 300 kb of the identified signals. In the case of loneliness (top panel) the results are from multi-trait GWAS, in the other cases the results are from linear mixed models

To identify genetic variation predisposing to loneliness, we performed a GWAS in the UK Biobank study (max sample *N* = 452,302) on the self-reported responses to three related questions ascertaining to perceived loneliness, frequency of social interactions, and ability to confide in someone. Results from these three GWAS were then combined using multi-trait GWAS (MTAG)^8^ into a single discovery sample, yielding an effective sample size of 487,647 individuals (see Methods). Across these data we estimated the heritability of loneliness to be 4.2% (S.E 0.02) and identified 15 genomic loci at genome-wide significance (*P* < 5 × 10^−8^, Fig. 1 Supplementary Data 1). A genetic risk score comprised of the 15 lead SNPs predicted loneliness in an independent set of 7556 individuals (*P* = 0.025).

**Fig. 2:**
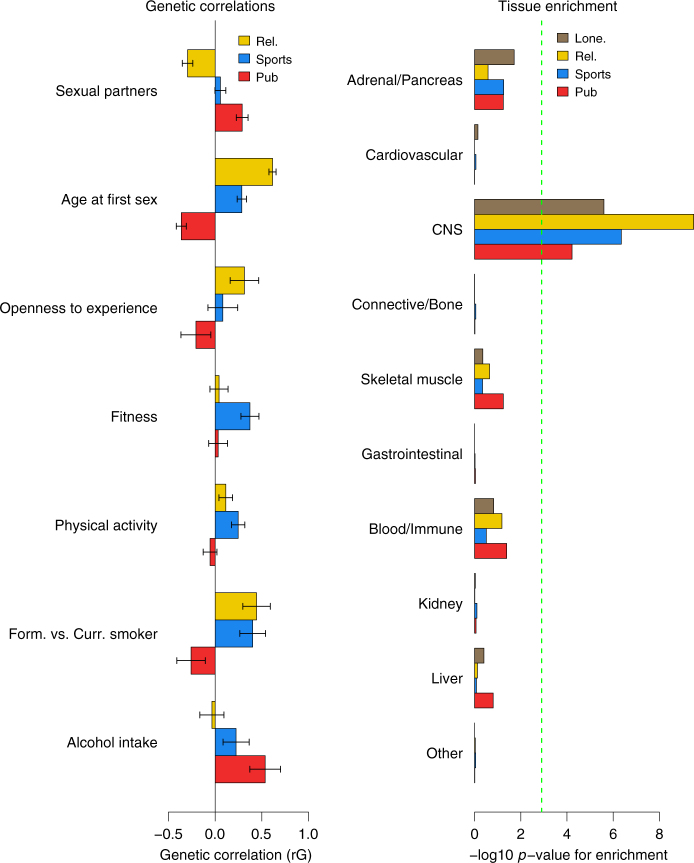
Genetic correlations and tissue enrichment results. Left: Genome-wide genetic correlations between regular participation in three different social activities–religious group (yellow), sports club /gym (blue), pub/social club (red). Right: Gene expression enrichment across genome-wide association results for the four social isolation and interaction traits, the green dotted line indicates Bonferroni corrected statistical  significance

By integrating gene expression and epigenetic data we sought to identify the relevant cell/tissue types implicated in the regulation of loneliness. We observed enrichment of association signals in regions surrounding genes that are preferentially expressed in several brain tissues (e.g., cerebellum, basal ganglia, and cortex; Fig. 2 Supplementary Table 1), in addition to enrichment for several epigenetic marks also in the basal ganglia, cortex and foetal brain (Supplementary Data 2). We next used FUSION^9^ to identify individual genes implicated by associated eQTL effects in GTEx brain tissues (Supplementary Data 3). Across 9178 transcripts in 9 tissue types, we identified 8 gene transcripts with expression levels putatively linked-to susceptibility to loneliness (*GPX1, C1QTNF4, C17orf58, MTCH2, BPTF, RP11-159N11.4, CRHR1-IT1* and *PLEKHM1*). *BPTF* encodes a transcription regulator that is highly expressed in foetal brain and is implicated in neurodegenerative diseases. *GPX1* and *MTCH2* are implicated in multiple metabolic pathways, including mitochondrial function.

### Estimating genetic overlap with other complex traits

Thirty-six complex traits exhibited significant genetic correlation with loneliness (Supplementary Data 4), including strong positive genetic overlap with neuroticism (*r*_g_ = 0.69, *P* = 2 × 10^−167^), and depressive symptoms (*r*_g_ = 0.84, *P* = 5.4 × 10^−153^), and strong negative genetic overlap with subjective well-being (*r*_g_ = −0.72, *P* = 1.2 × 10^−66^) and years of education (*r*_g_ = −0.33, *P* = 2.2 × 10^−43^). Given the high genetic correlation with depressive symptoms, we performed a sensitivity analysis by repeating the GWAS for loneliness excluding individuals with self-reported depression (*N* = 26,801). There was no appreciable change in test statistic across any of the 15 loci (Supplementary Data 1), median Chi-square value reduction ~13%), indicating that these loci do not influence loneliness via susceptibility to depression.

In addition to psychiatric and psychological traits, several anthropometric outcomes showed positive genetic correlations with loneliness (e.g., adult body mass index, BMI: *r*_g_ = 0.17, *P* = 4.4 × 10^−10^). We assessed the likely causal direction between BMI and loneliness by testing a bi-directional Mendelian randomisation (MR) framework, using genetic instruments and/or datasets derived independently from UK Biobank where necessary (Table 1). We found evidence supporting a positive causal effect of BMI on loneliness (*P*_IVW_ = 2.3 × 10^−6^), but not for loneliness on BMI (*P*_IVW_ = 0.58). We repeated these analyses using depressive symptoms, instead of loneliness, and found evidence supporting a positive bi-directional causal relationship with BMI (BMI-to-depressive symptoms: *P*_IVW_ = 0.017, depressive symptoms-to-BMI *P*_WM_ = 9.2 × 10^−4^). These MR analyses suggest direct causal links between social well-being and cardio-metabolic health, but do not preclude the possibility that both traits are causally downstream of shared biological pathways^10^. The observed heterogeneity in these models also suggests that the links between these traits are complex.

**Table 1:** Mendelian randomisation results

Exposure	Outcome	No. SNPs used	Model	Beta	SE	*P* value	Het. *P*-val
			**IVW**	**0.017**	**0.004**	**2.30** × 10^**−6**^	1
BMI (GIANT)	Loneliness (UKBB)	96	Egger’s	0.015	0.014	0.256	
			WM	0.021	0.007	1.43 × 10^−3^	
			**IVW**	**0.037**	**0.015**	**1.65** × 10^**−2**^	1
BMI (GIANT)	DS (23andMe)	96	Egger’s	0.042	0.046	0.365	
			WM	0.060	0.026	2.27 × 10^−2^	
Loneliness (UKBB)	BMI (UKBB)	13	IVW	3.571	0.532	1.86 × 10^−11^	2.14 × 10^−39^
			Egger’s	14.843	7.601	0.051	
			**WM**	**3.553**	**0.986**	**3.15** **×** **10**^**−4**^	
			IVW	0.281	0.040	1.20 × 10^−12^	3.30 × 10^−14^
DS (23andMe)	BMI (UKBB)	63	Egger’s	0.399	0.649	0.539	
			**WM**	**0.294**	**0.089**	**9.15** **×** **10**^**−4**^	
			**IVW**	**-0.043**	**0.079**	**0.585**	0.016
Lonely (UKBB)	BMI (GIANT)	15	Egger’s	1.689	0.913	0.064	
			WM	0.208	0.103	0.108	

Note: Results are reported for three different methods of MR–IVW (Inverse weighted variance), Egger’s and WM (weighted median). In each case the preferred model is highlighted in bold

*DS* depressive symptoms, *UKBB* UK Biobank, *GIANT* the genetic investigation of anthropometric traits

### Genetic discovery for engagement in social activities

To further explore potential biological mechanisms that confer susceptibility to social interactions, we performed additional GWAS analyses in the UK Biobank study for three further traits: regular attendance at a sports club or gym, pub or social club, and religious group (Fig. 1). The phenotypic overlaps between these traits are summarised in Supplementary Tables 2 and 3. Heritability estimates for these three traits ranged from 3.4% (sports club or gym) to 4.6% (religious group), placing them in the bottom 5% of heritability estimates for other complex traits, similar to other behavioural traits. We identified 38 genome-wide significant loci across the 3 traits (Fig. 1, Supplementary Data 5), of which 14 are correlated with previously reported signals for other behavioural/psychiatric traits (Supplementary Data 6). These traits demonstrated a partly shared genetic architecture–possibly indicating a shared propensity to social interactions (Supplementary Table 4).

We also observed trait-specific patterns of genetic correlations with other outcomes, concordant with reported non-genetic epidemiological correlations (Supplementary Data 4, Fig. 2). This trait specificity was supported by several of the individual loci (Supplementary Data 5); the most strongly associated variant for pub/social club attendance is a missense allele in the gene encoding alcohol dehydrogenase (*ADH1B-*rs1229984, *P*_pub_ = 4.2 × 10^−25^), which showed little or no association with sports club (*P* = 1.1 × 10^−2^) or religious group (*P* = 9.0 × 10^−1^) attendance. Furthermore, a recently reported signal for risk-taking propensity^11^ showed far stronger association with sports/gym attendance (*CADM2*-rs7627971, *P* = 5.8 × 10^−10^) than pub/social club (*P* = 3.0 × 10^−3^) or religious group (*P* = 3.9 × 10^−2^) attendance.

In contrast, several loci demonstrated pleiotropy across a range of complex traits, notably at the 1p22.2-*BARHL2* and 3p21.31-*CAMKV* regions. We identified two independent signals (*r*^2^ = 0.005, ~230Kb apart) near *BARHL2*, the first associated exclusively with pub/social club attendance (rs12759477, *P*_pub_ = 2.4 × 10^−13^, *P*_sport_ = 0.18, *P*_religious_ = 0.14), and the second with all three social interaction traits (rs699534, *P*_pub_ = 0.01, *P*_sport_ = 1.5 × 10^−6^, *P*_religious_ = 2.1 × 10^−10^). The latter signal is not correlated with a known signal for any other complex trait, whereas the former is partially correlated with reported signals for educational attainment (*r*^2^ = 0.08), chronotype (*r*^2^ = 0.27) and age at first sexual intercourse (*r*^2^ = 0.27). Similarly, at the 3p21.31-*CAMKV* region we identified two independent signals ~200 kb apart (*r*^2^ = 0.16). One signal, rs9837520, is associated primarily with religious group attendance (*P*_religious_ = 2.6 × 10^−8^, *P*_pub_ = 0.38, *P*_sport_ = 0.03) and is correlated with reported signals for inflammatory bowel disease (*r*^2^ = 1) and educational attainment (*r*^2^ = 0.75). The other signal, rs11712056, is associated with all three social interaction traits (all *P* < 6.1 × 10^−5^), and is correlated with reported signals for educational attainment (*r*^2^ = 1), resting heart rate (*r*^2^ = 0.12), HDL cholesterol (*r*^2^ = 0.43), blood pressure (*r*^2^ = 0.27), childhood ear infections (*r*^2^ = 0.18), age at menarche (*r*^2^ = 0.11) and age at first sex/birth (*r*^2^ = 0.35).

Finally, we explored which cell and tissue types were most relevant to the underlying biological processes regulating these social traits by performing partitioned LD score regression (see Methods). Genetic associations for all four traits were enriched for localisation to genes expressed in the central nervous system (*P*_min_ = 6 × 10^−5^, Fig. 2). When considering the 53 individual tissue types available in GTEx (Supplementary Data 2), significant (corrected *P*-value threshold 2.4 × 10^−4^) enrichments were seen for pub/social club attendance with the amygdala (brain) (*P* = 1.7 × 10^−4^) and for religious group attendance with the frontal cortex (*P* = 6.2 × 10^−6^), and in particular the anterior cingulate cortex (*P* = 1.1 × 10^−4^), which is located in the medial frontal lobe and is widely reported to regulate emotional self-control and problem-solving.

A limitation of our analysis was the absence of comparably sized independent replication studies to replicate associations with individual loci. This represents a challenge for genetic studies of complex traits with extremely large discovery datasets, such as UK Biobank, particularly for traits that are uncommonly measured. However, cumulative assessment of the polygenic risk score for loneliness in an unrelated sample that demonstrates the overall validity of our study design and analytical approach.

In summary, our findings highlight the specific genetic basis for social isolation and social interaction. We find evidence for shared genetic effects across social traits, in addition to more specific pathways that drive engagement in particular activities. Our findings also suggest a causal relationship between cardio-metabolic health and social isolation/mental health, an observation which warrants further investigation using other experimental approaches. Future studies should also aim to identify the potential mediators and modifiers that link mental health traits to obesity risk, such as eating behaviour, diet and physical activity. Finally, our findings provide a genetic resource for future studies to explore potential modifiable risk factors for social isolation.

## Methods

### Phenotype derivation in the UK Biobank study

The UK Biobank study includes half a million genotyped and phenotyped study participants and has been described extensively elsewhere.^6^. All participants provided informed written consent, the study was approved by the National Research Ethics Service Committee North West–Haydock, and all study procedures were performed in accordance with the World Medical Association Declaration of Helsinki ethical principles for medical research.

All traits analysed in this manuscript were derived from self-reported answers to questions directed via assessment centre touchscreen. We used data from three related questions assessing loneliness and social isolation–(1) *'*Do you often feel lonely?', to which individuals answered 'yes' (recorded as cases) or 'no' (controls), (2) A composite variable based on the questions 'Including yourself, how many people are living together in your household?' and *''*How often do you visit friends or family or have them visit you?' (cases were defined as those who lived alone and who indicated that they either never visited or had no friends or family outside their household; controls were defined as those who either did not live alone, or had friends who visited at least once a week) and (3) A variable representing quality of social interactions 'How often are you able to confide in someone close to you?' (cases were defined as those who answered 'Never or almost never', controls were defined as those who answered 'Almost daily'). Engagement in social activities was ascertained in response to the question 'Which of the following do you attend once a week or more often? (you can select more than one)' response options were: 'sports club or gym', 'pub or social club', 'religious group', 'adult education class', and 'other group activity'. Individuals with a positive response to the individual activity were coded as cases, all others as controls. Self-reported depression was in response to an interview question ascertaining doctor diagnosed illness.

### Genetic analysis in the UK Biobank study

We analysed data from the May 2017 release of imputed genetic data from UK Biobank, a resource extensively described elsewhere^12^. Given the reported technical error with non-HRC imputed variants, we focussed exclusively on the set of ~40 M imputed variants from the HRC reference panel. In addition to the quality control metrics performed centrally by UK Biobank, we defined a subset of 'white European' ancestry samples using a K-means clustering approach applied to the first four principle components calculated from genome-wide SNP genotypes. Individuals clustered into this group who self-identified by questionnaire as being of an ancestry other than white European were excluded. After application of QC criteria, a maximum of 452,302 individuals were available for analysis with genotype and phenotype data.

Association testing was performed using a linear mixed models implemented in BOLT-LMM^13^ to account for cryptic population structure and relatedness. Only autosomal genetic variants which were common (MAF > 1%), passed QC in all 106 batches and were present on both genotyping arrays were included in the genetic relationship matrix (GRM). Genotyping chip, age at baseline and genetic sex was included as a binary covariate in all models. Six GWAS models were run; loneliness (80,134 cases and 364,890 controls), rarely interacting with others (2426 cases, 286,524 controls), ability to confide (64,505 cases, 238,062 controls), regular participation in pub/social club (124,047 cases, 328,255 controls), sports club/gym (135,060 cases, 317,242 controls) and religious group (66,259 cases, 386,043 controls). We used MTAG software^8^ to combine the three GWAS datasets on perceived loneliness, living alone and ability to confide. MTAG is a recently described meta-analytical approach which enables to leverage variant discovery for  a target trait by borrowing statistic power from additional traits. MTAG is run using summary level GWAS data and can be applied to traits measured on different scales in overlapping (or the same) samples. Using this approach we increased the effective sample size of our primary loneliness variable from 445,024 to 487,647 (~10%) individuals. This composite trait was used for discovery, with statistically independent signals defined using 1 Mb clumping across all imputed variants with *P* < 5 × 10^−8^, an imputation quality score > 0.5 and MAF >1%.

### Replication data

Replication data for loneliness was available for an independent set of 7556 individuals previously reported and publicly available (https://www.med.unc.edu/pgc/results-and-downloads)^7^. Briefly, these data were derived from the Health and Retirement Study (HRS) using a 3-item questionnaire asking ‘How often do you feel that you lack companionship?' ‘How often do you feel left out?’ and ‘How often do you feel isolated from others?’. A linear measure was derived by summing the scores from all three questions, which was demonstrated to strongly correlate with the widely used UCLA loneliness scale. We then used the subsequent genetic dataset tested on this score in individuals of white European ancestry. Given the relatively small sample size and lack of individual level data, we estimated a combined allele score using the methods described below (causal inferences section). We accepted a directionally concordant result at *P* < 0.05 as evidence for replication.

### Causal inferences

Mendelian randomisation is an analytical method to infer the likely un-confounded causal relationship between an exposure trait and an outcome. The following models were run, each of which uses exposure/outcome data independent of discovery where required: (1) Assessing the causal effect of BMI (genetic risk score (*N* = 97) derived from GIANT^14^) on loneliness (composite MTAG variable from current UK Biobank study) and depressive symptoms (SSGC, https://www.thessgac.org/data). (2) Assessing the casual effect of loneliness (genetic risk score (*N* = 15) from current study) on BMI (GIANT^14^). (3) Assessing the casual effect of depressive symptoms (genetic risk score (*N* = 63) from 23andMe^15^ on BMI (UK Biobank).

In each case we performed inverse variance weighted, Egger’s and weighted median methods^16^. The inverse weighted median method is the  most powerful approach and is appropriate  when there is no evidence of heterogeneity amongst the SNPs, Egger’s is appropriate where there is evidence of directional pleiotropic effect and the weighted median approaches are prioritised in the presence of non-directional pleiotropy. We highlight the results from the Egger’s model where there was significant intercept (taken as evidence of directional pleiotropy), median weighted where there was significant heterogeneity (as assessed using the Cochran’s *Q* statistic); and inverse variance  weighted otherwise. A significance threshold of *P* = 0.0125 was set on the basis of four independent tests (i.e., = 0.05/4).

### Estimation of genetic correlations and heritability

Estimated heritability and genetic correlations (rg) were calculated between our four discovery traits (defined above) and over one hundred publicly available complex traits/diseases using the LD hub resource^17,18^. We additionally estimated genetic correlations against the following UK biobank traits: age at first sex, number of sexual partners, alcohol consumption and objectively derived physical activity/fitness^11^. Each was selected on the basis that it represents a well-established (and plausible) epidemiological correlate with one or more of the social interaction  traits.

### Gene expression enrichment testing

In order to identify which tissue types were most relevant to genes involved in social interaction/isolation, we applied LD score regression^19^ to specifically expressed genes (LDSC–SEG)^20^. Significance thresholds were set to reflect the number of tissues/traits tested. Enrichment testing of Epigenome Roadmap annotations was performed using the same framework described with the above approach. Physiological category enrichment was estimated using the same technique as previously described^19^. Individual eQTL analysis was performed using FUSION/TWAS, run using default settings (http://gusevlab.org/projects/fusion/)^9^. Only brain tissues from GTEx v6 were used, given the enrichment pattern observed from the LDSC-SEG analysis. A significance threshold of *P* = 5.45 × 10^−6^ was set to correct for 9178 genes tested across the 9 tissue types.

### Data availability

All UK Biobank data are available upon application (www.ukbiobank.ac.uk) and GWAS summary statistics can be downloaded from 10.17863/CAM.23511.

## Electronic supplementary material

Supplementary Information

Description of Additional Supplementary Files

Supplementary Data 1

Supplementary Data 2

Supplementary Data 3

Supplementary Data 4

Supplementary Data 5

Supplementary Data 6

## References

[1] Holt-Lunstad J, Smith TB (2012). Social relationships and mortality. Soc. Personal. Psychol. Compass.

[2] Victor C, Bowling A (2012). A longitudinal analysis of loneliness among older people in great britain. J. Psychol..

[3] Elovainio M (2017). Contribution of risk factors to excess mortality in isolated and lonely individuals: an analysis of data from the UK Biobank cohort study. Lancet Public Health.

[4] Holt-Lunstad J, Smith TB, Baker M, Harris T, Stephenson D (2015). Loneliness and social isolation as risk factors for mortality. Perspect. Psychol. Sci..

[5] Holt-Lunstad J, Smith TB, Layton JB (2010). Social relationships and mortality risk: a meta-analytic review. PLoS Med..

[6] Allen NE, Sudlow C, Peakman T, Collins R (2014). UK Biobank data: come and get it. Sci. Transl. Med..

[7] Gao J (2017). Genome-wide association study of loneliness demonstrates a role for common variation. Neuropsychopharmacology.

[8] Turley P (2018). Multi-trait analysis of genome-wide association summary statistics using MTAG. Nat. Genet..

[9] Gusev A (2016). Integrative approaches for large-scale transcriptome-wide association studies. Nat. Genet..

[10] Burgess S, Butterworth AS, Thompson JR (2016). Beyond Mendelian randomization: how to interpret evidence of shared genetic predictors. J. Clin. Epidemiol..

[11] Day FR (2016). Physical and neurobehavioral determinants of reproductive onset and success. Nat. Genet..

[12] Bycroft, C. et al. Genome-wide genetic data on ~500,000 UK Biobank participants. Preprint at http://www.biorxiv.org/content/early/2017/07/20/166298 (2017).

[13] Loh PR (2015). Efficient Bayesian mixed-model analysis increases association power in large cohorts. Nat. Genet..

[14] Locke AE (2015). Genetic studies of body mass index yield new insights for obesity biology. Nature.

[15] Hyde CL (2016). Identification of 15 genetic loci associated with risk of major depression in individuals of European descent. Nat. Genet..

[16] Bowden J, Davey Smith G, Haycock PC, Burgess S (2016). Consistent estimation in mendelian randomization with some invalid instruments using a weighted Median estimator. Genet. Epidemiol..

[17] Zheng J (2017). LD Hub: a centralized database and web interface to perform LD score regression that maximizes the potential of summary level GWAS data for SNP heritability and genetic correlation analysis. Bioinformatics.

[18] Bulik-Sullivan B (2015). An atlas of genetic correlations across human diseases and traits. Nat. Genet..

[19] Finucane HK (2015). Partitioning heritability by functional annotation using genome-wide association summary statistics. Nat. Genet..

[20] Finucane HK (2018). Heritability enrichment of specifically expressed genes identifies disease-relevant tissues and cell types. Nat. Genet..

