# Supplementary material for: Elucidating the genetic basis of social interaction and isolation
Source: Nat Commun. 2018 Jul 3;9:2457. doi: 10.1038/s41467-018-04930-1 (PMC6030100; doi:10.1038/s41467-018-04930-1)
Supplement: Supplementary file 1 — Supplementary Information [file 41467_2018_4930_MOESM1_ESM.pdf]

“Elucidating the genetic basis of social interaction and isolation”

Day *et al*

Supplementary Table 1 - LDSC-SEG GTEx enrichment

Name	Loneliness P	Pub P	Sport P	Religious
Brain Cerebellum	3.16E-03	3.93E-04	3.62E-02	1.64E-03
Brain Cerebellar Hemisphere	5.08E-03	2.54E-03	7.37E-02	4.83E-04
Brain Putamen (basal ganglia)	7.66E-03	8.80E-03	5.61E-04	2.48E-03
Brain Cortex	8.43E-03	2.55E-03	2.24E-02	7.77E-06
Brain Anterior cingulate cortex (BA24)	1.03E-02	1.61E-03	1.45E-02	1.08E-04
Brain Frontal Cortex (BA9)	1.61E-02	4.67E-03	1.77E-02	6.17E-06
Brain Caudate (basal ganglia)	1.71E-02	4.62E-04	1.29E-03	1.82E-03
Brain Hippocampus	1.73E-02	9.02E-03	1.69E-02	8.86E-03
Brain Substantia nigra	1.74E-02	1.12E-03	9.06E-02	3.62E-04
Brain Nucleus accumbens (basal ganglia)	2.18E-02	5.97E-04	1.28E-03	3.13E-04
Brain Hypothalamus	2.67E-02	2.83E-04	1.58E-02	7.56E-04
Brain Amygdala	7.94E-02	1.74E-04	7.79E-02	1.70E-03
Spleen	1.08E-01	6.06E-01	4.69E-01	2.51E-01
Ovary	1.45E-01	6.97E-01	9.94E-01	3.03E-01
Brain Spinal cord (cervical c-1)	1.80E-01	1.69E-03	4.50E-02	1.48E-02
Adrenal Gland	2.17E-01	7.14E-01	9.87E-01	7.89E-01
Thyroid	2.31E-01	9.98E-01	8.72E-01	7.60E-01
Small Intestine Terminal Ileum	2.34E-01	6.31E-01	9.85E-01	8.42E-01
Prostate	2.69E-01	6.62E-01	9.92E-01	4.77E-01
Stomach	3.82E-01	1.56E-01	9.98E-01	7.14E-01
Colon Transverse	3.89E-01	7.79E-01	5.36E-01	6.48E-01
Bladder	3.91E-01	9.83E-01	9.95E-01	9.05E-01
Pituitary	4.30E-01	3.99E-01	7.56E-01	2.03E-02
Vagina	4.45E-01	9.97E-01	6.04E-01	1.62E-01
Esophagus Muscularis	5.69E-01	7.80E-01	9.62E-01	5.05E-01
Esophagus Mucosa	5.94E-01	8.57E-01	6.89E-01	6.28E-01
Uterus	6.17E-01	8.43E-01	5.96E-02	4.31E-01
Minor Salivary Gland	6.30E-01	9.53E-01	9.52E-01	9.01E-01
Testis	6.32E-01	2.73E-01	5.49E-01	1.05E-01
Cervix Ectocervix	6.33E-01	9.97E-01	9.85E-01	7.40E-01
Muscle Skeletal	6.42E-01	8.30E-02	8.83E-01	9.28E-02
Cells EBV-transformed lymphocytes	6.44E-01	4.75E-01	3.96E-01	1.05E-01
Heart Left Ventricle	7.30E-01	6.50E-01	6.24E-01	4.42E-01
Colon Sigmoid	7.46E-01	8.52E-01	4.61E-01	8.11E-01
Nerve Tibial	7.51E-01	2.29E-01	7.63E-01	7.02E-01
Cervix Endocervix	7.68E-01	8.52E-01	9.98E-01	4.56E-01
Esophagus Gastroesophageal Junction	7.76E-01	5.60E-01	8.97E-01	5.53E-01
Heart Atrial Appendage	7.80E-01	5.51E-01	8.24E-01	9.71E-01
Lung	7.90E-01	6.10E-01	9.65E-01	9.70E-01
Kidney Cortex	8.11E-01	4.64E-01	9.97E-01	9.10E-01
Skin Not Sun Exposed (Suprapubic)	8.21E-01	9.99E-01	9.48E-01	6.77E-01
Liver	8.55E-01	2.28E-01	9.98E-01	4.10E-01
Skin Sun Exposed (Lower leg)	8.76E-01	9.93E-01	8.09E-01	4.09E-01
Fallopian Tube	9.05E-01	6.69E-01	8.19E-01	9.44E-01
Whole Blood	9.09E-01	7.76E-01	9.86E-01	4.93E-01
Adipose Visceral (Omentum)	9.59E-01	8.70E-01	4.24E-01	1.00E+00
Artery Aorta	9.75E-01	8.10E-01	8.20E-01	9.13E-01
Breast Mammary Tissue	9.78E-01	9.90E-01	8.39E-01	8.04E-01
Artery Tibial	9.85E-01	7.57E-01	8.24E-01	5.65E-01
Adipose Subcutaneous	9.85E-01	8.34E-01	8.00E-01	9.65E-01
Cells Transformed fibroblasts	9.87E-01	3.45E-01	8.80E-01	9.89E-01
Pancreas	9.89E-01	4.94E-01	9.82E-01	9.54E-01
Artery Coronary	9.99E-01	9.85E-01	2.76E-01	8.62E-01

Supplementary Table 2 - Phenotypic distribution

Attend a Pub or Social Club	Attend Sports activity	Attend Religious groups	Percentage of total (%)	Loneliness		
				Yes	No	% Yes
Yes	Yes	Yes	0.8	537	3,111	17.3
		No	7.8	5,141	29,432	17.5
	No	Yes	1.7	1,298	6,320	20.5
		No	17.2	14,149	62,208	22.7
No	Yes	Yes	3.3	2,106	12,657	16.6
		No	18.1	12,534	67,814	18.5
	No	Yes	8.8	6,604	32,601	20.3
		No	42.4	37,765	150,747	25.1

Supplementary Table 3 - Phenotypic associations

Social group	Odds ratio for Loneliness	Standard Error	95% CI	p-value
Pub or Social Club (raw)	0.93	7.9×10^{-3}	0.92 – 0.95	3.8×10^{-17}
Pub or Social Club (adjusted)	1.00	8.8×10^{-3}	0.99 – 1.02	0.76
Sports group (raw)	0.75	6.4×10^{-3}	0.74 – 0.76	3.9×10^{-243}
Sports group (adjusted)	0.73	6.3×10^{-3}	0.71 – 0.74	4.6×10^{-298}
Religious group (raw)	0.87	9.5×10^{-3}	0.85 – 0.88	1.7×10^{-39}
Religious group (adjusted)	0.85	9.5×10^{-3}	0.83 – 0.87	2.2×10^{-47}
The adjusted model is adjusted for age and sex, as well as attendance at the other two social groups.				

Supplementary Table 4 – Genetic correlations between phenotypes relating to social contact.

Trait 1	Trait 2	rG	SE	z	p
Lonely	Pub or social club	0.0085	0.034	0.25	0.80
Lonely	Religious group	-0.284	0.036	-8.00	1.20E-15
Lonely	Sports group	-0.314	0.032	-9.95	2.53E-23
Pub or social club	Religious group	-0.347	0.037	-9.29	1.57E-20
Pub or social club	Sports group	0.079	0.033	2.39	0.02
Religious group	Sports group	0.306	0.032	9.52	1.77E-21
