# Supplementary material for: Elucidating the genetic basis of social interaction and isolation
Source: Nat Commun. 2018 Jul 3;9:2457. doi: 10.1038/s41467-018-04930-1 (PMC6030100; doi:10.1038/s41467-018-04930-1)
Supplement: Supplementary file 2 — Description of Additional Supplementary Files [file 41467_2018_4930_MOESM2_ESM.pdf]

Description of Additional Supplementary Files

File Name: Supplementary Data 1
Description: GWAS Loneliness Loci. Loci identified using MTAG analysis, and the results for associations with each of the phenotypes used in the MTAG analysis.

File Name: Supplementary Data 2
Description: LDSC-SEG EpiRoadmap. Tissue enrichment analysis.

File Name: Supplementary Data 3
Description: FUISON eQTL Results. Gene based tests, based on eQTL data from tissues highlighted by tissue enrichment analysis.

File Name: Supplementary Data 4
Description: Genetic Correlations. Correlations between four social phenotypes and other health related phenotypes.

File Name: Supplementary Data 5
Description: Identified Social Loci. Loci identified for the three social interaction phenotypes.

File Name: Supplementary Data 6
Description: GWAS catalogue results. Previously identified associations for the SNPs highlighted by the social analysis.
